# Molecular detection and characterization of *Babesia bovis*, *Babesia bigemina, Theileria* species and *Anaplasma marginale* isolated from cattle in Kenya

**DOI:** 10.1186/s13071-015-1106-9

**Published:** 2015-09-30

**Authors:** Paul Franck Adjou Moumouni, Gabriel Oluga Aboge, Mohamad Alaa Terkawi, Tatsunori Masatani, Shinuo Cao, Ketsarin Kamyingkird, Charoonluk Jirapattharasate, Mo Zhou, Guanbo Wang, Mingming Liu, Aiko Iguchi, Patrick Vudriko, Adrian Patalinghug Ybanez, Hisashi Inokuma, Rika Shirafuji-Umemiya, Hiroshi Suzuki, Xuenan Xuan

**Affiliations:** National Research Center for Protozoan Diseases, Obihiro University of Agriculture and Veterinary Medicine, Obihiro, Hokkaido 080-8555 Japan; Department of Public Health, Pharmacology and Toxicology, Faculty of Veterinary Medicine, University of Nairobi, Kangemi, Nairobi 00625 Kenya; United Graduate School of Veterinary Sciences, Gifu University, Gifu, Gifu 501-1193 Japan; Department of Veterinary Clinical Sciences, Obihiro University of Agriculture and Veterinary Medicine, Obihiro, Hokkaido 080-8555 Japan

**Keywords:** Epidemiology, PCR, *Babesia*, *Theileria*, *Anaplasma*, Cattle, Kenya

## Abstract

**Background:**

Infections with *Babesia bovis*, *Babesia bigemina, Theileria* species and *Anaplasma marginale* are endemic in Kenya yet there is a lack of adequate information on their genotypes. This study established the genetic diversities of the above tick-borne hemoparasites infecting cattle in Kenya.

**Methods:**

Nested PCR and sequencing were used to determine the prevalence and genetic diversity of the above parasites in 192 cattle blood samples collected from Ngong and Machakos farms. *B. bovis* spherical body protein 4, *B. bigemina* rhoptry-associated protein 1a, *A. marginale* major surface protein 5, *Theileria* spp. 18S rRNA, *T. parva* p104 and *T. orientalis* major piroplasm surface protein were used as the marker genes.

**Results:**

*B. bovis*, *B. bigemina*, *T. parva*, *T. velifera*, *T. taurotragi*, *T. mutans* and *A. marginale* were prevalent in both farms, whereas *T. ovis, Theileria* sp*.* (buffalo) and *T. orientalis* were found only in Ngong farm. Co-infections were observed in more than 50 % of positive samples in both farms. *Babesia* parasites and *A. marginale* sequences were highly conserved while *T. parva* and *T. orientalis* were polymorphic. Cattle-derived *T. parva* was detected in Machakos farm. However, cattle and buffalo–derived *Theileria* were detected in Ngong farm suggesting interactions between cattle and wild buffaloes. Generally, the pathogens detected in Kenya were genetically related to the other African isolates but different from the isolates in other continents.

**Conclusions:**

The current findings reaffirm the endemicity and co-infection of cattle with tick-borne hemoparasites, and the role of wildlife in pathogens transmission and population genetics in Kenya.

**Electronic supplementary material:**

The online version of this article (doi:10.1186/s13071-015-1106-9) contains supplementary material, which is available to authorized users.

## Background

Babesiosis, theileriosis and anaplasmosis are important tick-borne diseases of cattle worldwide including Kenya. Babesiosis and theileriosis are caused by the protozoan parasites, *Babesia* and *Theileria* respectively while anaplasmosis is caused by rickettsial organism known as *Anaplasma*. In Africa including Kenya, bovine babesiosis is caused by *Babesia bovis* and *B. bigemina* and the disease is transmitted by *Rhipicephalus* ticks [1]. Though, *B. bigemina* is more widespread, *B. bovis* infection is the most critical and fatal because of its neurological symptoms [2].

Bovine theileriosis is another tick-borne disease found in many African countries. Tropical theileriosis and East Coast fever (ECF) are the most severe forms of the disease. Tropical theileriosis, caused by *T. annulata* and transmitted by *Hyalomma* ticks is distributed in Northern Africa. ECF is probably the most important tick-borne disease in Eastern, Central and Southern Africa. The disease is caused by *Theileria parva* and transmitted mainly by *Rhipicephalus appendiculatus. T. parva* natural host is the African Cape buffalo (*Syncerus caffer*), which serves as source of infection for cattle. Some variants of the parasite are transmitted solely from buffalo to cattle whereas others can spread from cattle to cattle. The other *Theileria* species reported in Africa are *T. mutans*, *T. taurotragi*, *T. sergenti/buffeli/orientalis* (referred to as *T. orientalis* complex) and *T. velifera.* These species are considered to be either less pathogenic or not pathogenic at all, and only cause benign, moderate to asymptomatic theileriosis [3]. For bovine anaplasmosis, *Anaplasma marginale* is one of the causative agents and this pathogen is transmitted biologically, by approximately twenty tick species, and mechanically by biting flies and blood-contaminated fomite [4].

*B. bovis*, *B. bigemina, T. parva, T. annulata* and *A. marginale* cause mortalities and morbidities leading to losses in production of milk, meat, and other livestock by-products. Consequently, they cause severe economic losses to livestock farmers involved in dairy and beef production in tropical and sub-tropical regions [5]. In Kenya, theileriosis, anaplasmosis and babesiosis are ranked among the most common causes of economic losses in dairy and beef industry [6–8]. The diagnosis of these diseases in Kenyan cattle have relied mostly on clinical signs [6], microscopic examination of blood smears [6, 9–12] and antibody detection [12–14]. A few studies employing molecular methods (polymerase chain reaction (PCR), reverse line blot hybridization (RLB), real time PCR) and genome sequencing have been exploited in Kenya [15–18]. However, these studies have been limited to a few tick-borne diseases with most of the studies lacking adequate information on their genotypes or even knowledge on their molecular epidemiology, which is critical for the control and prevention of these diseases.

Therefore, this study was done with the main objective of determining and understanding the genetic diversities and molecular epidemiology of some species of *Babesia, Theileria* and *Anaplasma* pathogens infecting cattle in Kenya. In particular, blood samples of cattle raised in farms located in Machakos and Ngong districts of Kenya were screened for specific target genes of *B. bovis*, *B. bigemina, Theileria* spp. and *A. marginale.* The sequences generated from these target genes were used to confirm the identity of the pathogens and establish phylogenies to aid in the understanding of their molecular epidemiology in Kenya in relation to other regions of the world.

## Methods

### Study areas and blood samples collection

Two separate dairy cattle farms were investigated in Kenya with one farm located in Ngong district of Kajiado County and the other farm is located in Machakos district of Machakos County. The farm in Ngong district (1° 22′S, 36° 38′E) lies 22 km Southwest of Nairobi whereas the other one in Machakos district (1° 14′S, 37° 23′E) lies 63 km Southeast of Nairobi. The average annual temperatures and rainfalls are 16.7 °C, 865 mm and 19.0 °C, 830 mm for Ngong and Machakos, respectively [19]. Cattle in both farms are kept under a semi-extensive system, characterized by free grazing on pastures. The cattle were kept under semi-enclosed system at night and allowed to graze on pastures where the animals mixed with Masaai cattle grazing in the same area. The grazing together with Masaai cattle was particularly seen in case of cattle kept in Ngong farm.

A total of 154 and 38 blood samples were collected in EDTA-vacutainer tubes from cattle in Ngong and Machakos farms, respectively during a cross-sectional survey done in August 2011. The samples were collected from randomly selected male and female crossbred cattle including adults and yearlings; all of which were apparently healthy. The samples were then transported on ice to the Central Veterinary Laboratory in Nairobi, Kenya and stored at −20 °C prior to DNA extraction.

### Ethical statement

The managers of surveyed farms were informed about the study and gave their approval for the sampling of cattle. All the procedures were carried out according to ethical guidelines for the use of animal samples permitted by Obihiro University of Agriculture and Veterinary Medicine (Permit for animal experiment: 26–73, 26–72; DNA experiment: 1219–3; Pathogen: 201210–5; 201206–5).

### Extraction of hemoparasites DNA

The genomic DNA was extracted at the Central Veterinary Laboratory in Nairobi, Kenya using a commercial DNA extraction kit according to the manufacturer’s instructions (QIAamp DNA Blood Mini-Kit, Germany). The extracted DNA samples were transported to the National Research Center for Protozoan diseases in Obihiro, Japan where they were stored at −30 °C pending further genetic analysis.

### Hemoparasites DNAs-detection by nPCR

Specific primers targeting *B. bovis* spherical body protein-4 (SBP-4)*, B. bigemina* rhoptry-associated protein-1a (RAP-1a), *Theileria* spp. 18S rRNA and *A. marginale* major surface protein 5 (Msp5) genes were used to amplify the respective genes by using previously described nPCRs [20–22]. Initial PCR amplifications were done in a 10 μl-reaction mixture having 1 μl of DNA template, 1 μl (10 μM) of each primers, 1μlof 10x Ex buffer, 1 μl of dNTP (200 μM each), 0.1 μl of Ex Taq polymerase (Takara, Japan) and 4.9 μl of double distilled water. A nested PCR was done using 1 μl of DNA template obtained from the first PCR amplification. Primers and thermocycling conditions were as described previously (Table 1) except for *A. marginale* in which a touch down PCR was done. The following samples were used as positive controls; DNAs of *B. bigemina* (Argentina strain)*, B. bovis* (Texas strain), *T. parva (*Muguga G6,ILRI), *T. annulata* (Ankara C9,Edinburgh University), cattle DNA sample positive for *T. orientalis* and *A.marginale*-Msp5 plasmid [22]. Double distilled water was used as a negative control. The nPCR products were electrophoresed, stained with ethidium bromide and then visualized under UV transilluminater.

**Table 1:** Sequences of primers set used for detection of hemoparasites DNAs

Pathogen	Assays	Oligonucleotide sequences (5' > 3')	Product size (bp)	Reference
Target gene				
*B. bovis* SBP-4	PCR	AGTTGTTGGAGGAGGCTAAT	907	[21]
		TCCTTCTCGGCGTCCTTTTC		
	nPCR	GAAATCCCTGTTCCAGAG	503	
		TCGTTGATAACACTGCAA		
*B. bigemina* RAP-1a	PCR	GAGTCTGCCAAATCCTTAC	879	
		TCCTCTACAGCTGCTTCG		
	nPCR	AGCTTGCTTTCACAACTCGCC	412	
		TTGGTGCTTTGACCGACGACAT		
	semi nPCR	GAGTCTGCCAAATCCTTAC	690	
		TTGGTGCTTTGACCGACGACAT		
*Theileria* spp*.* 18S rRNA	PCR	GAAACGGCTACCACATCT	778	[20]
		AGTTTCCCCGTGTTGAGT		
	nPCR	TTAAACCTCTTCCAGAGT	581	
		TCAGCCTTGCGACCATAC		
*T. parva* p 104	PCR	ATTTAAGGAACCTGACGTGACTGC	496	[23]
		TAAGATGCCGACTATTAATGACACC		
	nPCR	GGCCAAGGTCTCCTTCAGATTACG	277	
		TGGGTGTGTTTCCTCGTCATCTGC		
*T. orientalis* MPSP	PCR	CTTTGCCTAGGATACTTCCT	776	[24]
		ACGGCAAGTGGTGAGAACT		
*A. marginale* Msp5	PCR	GTGTTCCTGGGGTACTCCTATGTGAACAAG	547	[22]
		AAGCATGTGACCGCTGACAAACTTAAACAG		
	nPCR	AAGCACATGTTGGTAATATTCGGCTTCTCA	195	
		AATTCTCGCATCAAAAGACTTGTGGTACTC		

*Note*: The primers sets forSBP-4, 18S rRNA, p104 and MPSP genes were used for detection of corresponding pathogens and the products of the last amplification served as template for genetic characterisation. With regard to BbigRAP-1a and Msp5, PCR and nPCR primers were used in pathogen detection, however for genetic characterization, amplicons from a semi-nPCR (BbigRAP-1) and from the first PCR (Msp5) were used

To detect co-infection with *T. parva* and *T. orientalis* complex, samples positive for *Theileria* spp. 18S rRNA were further amplified by nPCR using primers targeting *T. parva* p104 (p104) gene [23] for *T. parva* and those targeting *T. orientalis* major piroplasm surface protein (MPSP) gene [24] for *T. orientalis*.

### Sequencing of the hemoparasites DNAs

All *Theileria* spp. positive samples and randomly selected positive samples of *B. bovis, B. bigemina, T. parva, T. orientalis* complex and *A. marginale* (three samples for each parasite per farm) were used as templates for genetic characterization of the hemoparasites. Nested PCR amplicons of *Theileria* spp. 18S rRNA, *B. bovis* SBP-4 and *T. parva* p104; PCRs amplicons of *A. marginale* Msp5 and *T.orientalis* MPSP; and the products of *B. bigemina* RAP-1a-semi-nPCR (Table 1) were purified by using QIAquick Gel Extraction Kit (QIAGEN GmbH, Germany). The *Theileria* spp. 18S rRNA amplicons were sequenced with the nPCR primers. DNA sequences with heterozygous base-calling were analyzed using Mixed Sequence Reader web-based program [25] and identified as two distinct sequences. The other purified DNA templates were first sequenced with the amplification primers to identify heterozygous base-calling positions and then cloned in pGEM-T Easy Vector (Promega, USA). Initially, two positive clones per template were randomly selected and sequenced with pGEM-T Easy Vector-primers (pUC/M13). When the sequences of the clones did not include all the genotypes identified during direct sequencing, other two clones from the same template were sequenced. All sequencing analysis assays were performed using the Dye Terminator Cycle Sequencing Kit (Applied Biosystems, USA) and an ABI PRISM 3100 genetic analyzer (Applied Biosystems, USA).

### Blast analysis, sequence alignment and phylogenetic analysis

The sequenced DNAs were analyzed by BLASTn tool of NCBI GenBank database. The correct species identity was established by comparing the query sequences with those available in the GenBank database. Species confirmation was done when the closest BLASTn match has a 98 %-100 % identity to the homologues found in the GenBank. DNA sequences identities were also computed using the pairwise alignment by EMBOSS NEEDLE software [26]. Multiple sequence alignments were performed using MUSCLE and GUIDANCE algorithms [27]. Phylogenetic analyses were inferred by the maximum likelihood method using MEGA version 6 software [28].

### Statistical analysis

Proportions of DNA samples positive for respective hemoparasites per farm were computed and a comparison of pathogen prevalence was done using the chi-square test employing the EPI INFO™ software (CDC, USA, version 7.1.1) and VassarStats [29]. Statistically significant differences were determined at *P* < 0.05.

### Nucleotide sequences accession numbers

The nucleotide sequences of all the genes sequenced are available in the GenBank of the NCBI database under the accession numbers outlined in Table 2.

**Table 2:** Accession numbers of DNA sequences deposited in GenBank for the hemoparasites detected in this study

Parasite isolate	Target genes	Accession numbers	Sequence length (bp)
*B. bovis*	SBP-4	KP347555	521
		KP347556	521
		KP347557	521
*B. bigemina*	RAP-1a	KP347558	690
		KP347559	690
		KP893330	690
*A. marginale*	Msp5	KP347553	576
		KP347554	576
*Theileria* spp*.*	18S rRNA	KP347567	486
		KP347568	493
		KP347569	497
		KP347570	515
		KP347571	512
		KP347572	512
		KP347573	488
		KP347574	514
		KP347575	469
*T. parva*	p104	KP347564	278
		KP347565	278
		KP347566	278
*T. orientalis*	MPSP	KP347560	776
		KP347561	776
		KP347562	776
		KP347563	776

## Results

### Detection of hemoparasites in cattle by nPCR

A total of 192 blood samples (154 in Ngong and 38 in Machakos) were analyzed by nPCR to be able to detect infection of cattle with *B. bovis, B. bigemina*, *Theileria* spp. and *A. marginale*. Of these samples, at least one of the hemoparasites named above was detected in 135 (70 %) samples. In Ngong farm, 110 (71 %) samples had hemoparasites DNAs with *B. bigemina* (65 samples- 42.2 %) being more prevalent than *B. bovis* (19 samples-12.3 %). On the other hand, *Theileria* spp. and *A. marginale* DNAs were detected in 52 (33.8 %) and 50 (32.5 %) blood samples, respectively. Overall, 25 (66 %) samples were positive for at least one hemoparasite in Machakos. In particular, 9 (23.7 %) samples were positive for *B. bovis* while *B. bigemina* was detected in 5 (13.2 %) samples. *Theileria* spp. and *A. marginale* were detected in 15 (39.5 %) and 6 (15.8 %) samples, respectively. There was statistically significant difference in prevalences observed in the two farms (*P* < 0.05) for *B. bigemina* and *A. marginale*. Individual results for each parasite are shown in an additional file [see Additional file 1].

### Identification of *Theileria* species

Genetic analysis of the sequenced *Theilera* spp.-18S rRNAs revealed homologues that belonged to seven distinct *Theileria* species including *T. parva*, *T. taurotragi*, *T. mutans*, *T. velifera, T.orientalis* complex, *T. ovis* and *Theileria* sp. (buffalo) (Table 3). In particular, *T. parva*, *T. taurotragi*, *T. mutans* and *T. velifera* were detected in samples from cattle in both farms while *T. orientalis* complex, *T. ovis* and *Theileria* sp*.* (buffalo) were detected in Ngong farm-cattle only. Co-infections with *T. parva* and *T. taurotragi*, *T.mutans* and *T. taurotragi* were observed in some blood samples from Ngong farm-cattle. Other samples with *T. parva* and *T. velifera* co-infections were seen in the same farm. In contrast, co-infections with *T. taurotragi* and *T. velifera* were detected in cattle from Machakos farm only. All the samples that were positive for *T. parva* and *T. orientalis* complex 18S rRNAs were also positive for *T. parva* p104 and *T. orientalis* MPSP genes, respectively. Thirty five samples from Ngong farm and 11 samples from Machakos farm that were negative for *T. parva* 18S rRNA were found to be positive for *T. parva* p104 DNA. *T. parva* was the most prevalent with the parasite DNA being detected in 46 (29.9 %) and 12 (31.6 %) samples from cattle in Ngong and Machakos farms, respectively. Infections with *T. velifera, T. taurotragi* and *T. mut*ans were also observed though less frequently. Few cases of *T.orientalis* complex, *T. ovis* and *Theileria* sp*.* (buffalo) infections were detected. Detailed results of *Theileria* species identification are shown in an additional file [see Additional file 2].

**Table 3:** Identification of *Theileria* species by BLASTn analysis of the 18S rRNA sequences of the isolates from cattle in Kenya

Accession number	Highest Blastn match	Accession number of match	% identity
KP347567	*T. mutans*	AF078815	99
KP347568	*T. orientalis*	AB520955	100
	*T. sergenti*	JQ723015	
	*T. buffeli*	DQ287959	
	*Theileria* sp*.* JW-2014	KJ020546	
KP347569	*T. ovis*	KM924444	100
KP347570	*T. parva*	HQ684067	100
KP347571	*T. taurotragi*	L19082	99
KP347572	*T. taurotragi*	L19082	100
KP347573	*T. taurotragi*	L19082	98
KP347574	*T. velifera*	JN572705	100
KP347575	*Theileria* sp.(buffalo)	HQ895982	100

### Mixed infections with *Babesia*, *Theileria* and *Anaplasma*

More than half of the positive samples were infected with at least two hemoparasites, which generally belonged to different genus. Twenty nine different types of mixed infections were seen in Ngong farm with some cattle having up to five pathogens co-infecting the same cattle (Table 4). In Machakos farm, 11 different types of mixed infections were seen with up to three pathogens simultaneously being detected in some samples (Table 5). Single infections with *B. bovis*, *T. velifera* and *A. marginale* were observed in both farms, while single infections with *B. bigemina* and *T. parva* were seen in Ngong farm only. In most cases, mixed infections rather than single infection were detected for all hemoparasites in both farms except for *B. bovis* for which most of positive samples in Machakos farm were single infections (Table 5).

**Table 4:** Tick-borne hemoparasites detected in cattle (*n* = 154) from Ngong-farm in Kenya

Pathogen species detected	Positive cattle (%)
One pathogen	
*B. bovis*	8 (5.2)
*B. bigemina*	22 (14.3)
*T. parva*	3 (1.9)
*T. velifera*	2 (1.3)
*A. marginale*	12 (7.8)
Two pathogens	
*B.bigemina* + *T. parva*	3 (1.9)
*B.bigemina* + *A. marginale*	11 (7.1)
*A. marginale + T. parva*	1 (0.6)
*A. marginale + Theileria* sp. (buffalo)	1 (0.6)
*A. marginale + T. taurotragi*	1 (0.6)
*B.bovis* + *B. bigemina*	2 (1.3)
*B.bovis* + *A. marginale*	1 (0.6)
*T. parva* + *T.velifera*	5 (3.2)
*T. parva + T.mutans*	1 (0.6)
Three pathogens	
*B.bovis* + *B. bigemina* + *A. marginale*	2 (1.3)
*B. bovis + T. parva + T. ovis*	1 (0.6)
*B.bigemina* + *T. parva + T. mutans*	3 (1.9)
*B. bigemina + T. parva + T. velifera*	4 (2.6)
*B. bigemina + T. parva + T. ovis*	1 (0.6)
*B. bigemina + T. taurotragi + T. mutans*	1 (0.6)
*B. bigemina + T. taurotragi + T. parva*	4 (2.6)
*B.bigemina* + *A. marginale* + *T. taurotragi*	1 (0.6)
*B.bigemina* + *A. marginale* + *T. parva*	1 (0.6)
*A. marginale + T. parva + T. taurotragi*	2 (1.3)
*A. marginale + T. parva + T. velifera*	4 (2.6)
*A. marginale + T. parva + T. orientalis*	2 (1.3)
*A. marginale + T. parva + T. mutans*	1 (0.6)
Four pathogens	
*B. bigemina + A. marginale + T. parva + T. taurotragi*	1 (0.6)
*B. bigemina + A. marginale + T. parva + T. ovis*	1 (0.6)
*B. bigemina + A. marginale + T. parva + T. mutans*	3 (1.9)
Five pathogens	
*B.bovis + B. bigemina + A. marginale + T. parva + T.orientalis*	1 (0.6)
*B.bovis + B. bigemina + A. marginale + T. parva + T. mutans*	2 (1.3)
*B.bovis + B. bigemina + A. marginale + T. parva + T. taurotragi*	1 (0.6)
*B.bovis + B. bigemina+ + A. marginale + T. parva + T. velifera*	1 (0.6)
Total	110 (71.4)

**Table 5:** Tick-borne hemoparasites detected in cattle (*n* = 38) from Machakos-farm in Kenya

Pathogen species detected	Positive cattle (%)
One pathogen	
*B. bovis*	6 (15.8)
*T. velifera*	1 (2.6)
*A. marginale*	3 (7.9)
Two pathogens	
*B.bigemina* + *T. parva*	1 (2.6)
*B.bigemina* + *T. taurotragi*	1 (2.6)
*B.bovis* + *B. bigemina*	1 (2.6)
*T. parva* + *T. velifera*	2 (5.3)
*T. taurotragi* + *T.velifera*	1 (2.6)
*T. parva + T. taurotragi*	1 (2.6)
Three pathogens	
*B. bovis + T. parva + T. mutans*	2 (5.3)
*B. bigemina + T. parva + T. velifera*	2 (5.3)
*A. marginale + T. parva + T. velifera*	2 (5.3)
*A. marginale + T. parva + T. mutans*	1 (2.6)
*T. parva + T. taurotragi + T. velifera*	1 (2.6)
Total	25 (65.8)

### Blast analysis and sequence alignment

To establish the genotypes of these tick-borne pathogens, sequences of SBP-4, RAP-1a, Msp5, p104 and MPSP genes found in *B. bovis, B. bigemina, A. marginale, T. parva* and *T. orientalis*, respectively were genetically characterized. The identity values among the nucleotides sequences of *B. bovis* SBP-4 determined in this study [KP347555, KP347556 and KP347557] ranged from 99.6 to 99.8 %. Blastn analysis revealed that two Kenyan *B. bovis* isolates [KP347555 and KP347557] shared 99 % nucleotide sequence identity with an isolate from Egypt [KF192807], while the other Kenyan isolate [KP347556] shared 100 % sequence identity with the isolates from South Africa [KF626630 and AB569303] and Ghana [AB569301]. The multi-sequence alignment of *B. bovis* SBP-4 amino acid residues revealed that the Kenyan isolates contained additional amino acid residues and a specific pattern of substitutions unique to isolates obtained from African cattle (Table 6). For *B. bigemina*, the identities among Kenyan RAP-1a sequences ranged from 99.6 to 99.9 %. A further blastn analysis of RAP-1a sequences revealed that the *B. bigemina* Kenyan isolates shared between 99 % and 100 % nucleotide identities with the sequence of an Egyptian isolate [KF192811]. The *A. marginale* Msp5 sequences identified in this study [KP347553 and KP347554] shared 99.8 % identity with each other and were homologous to the sequences of isolates from Australia [CP006847], the Philippines [AB704328] and China [EF546443].

**Table 6:** Alignments of amino acid substitutions observed in the SBP-4 gene of Kenya *B. bovis* isolates in comparison with sequences from other geographic areas

*Babesia bovis* Spherical body protein 4 (SBP-4) gene																								
Isolate			1	1	1	1	1	1	1	1	1	1	1	1	1	1	1	1	2	2	2	2	2	2
			1	1	2	3	3	3	3	3	3	3	4	4	5	7	7	9	0	2	2	5	6	7
GenBank ID	Country	Source	0	8	9	2	3	4	5	6	7	8	2	8	9	2	3	2	4	1	8	4	3	0
XM_001610418	USA	Cattle	E	T	G	**-**	**-**	**-**	**-**	**-**	**-**	A	D	I	D	V	S	D	F	L	A	F	T	A
AB569300	Brazil	Cattle	*	*	*	**-**	**-**	**-**	**-**	**-**	**-**	*	*	*	*	*	*	*	*	*	*	*	*	*
AB569302	Mongolia	Cattle	K	*	*	**-**	**-**	**-**	**-**	**-**	**-**	*	*	*	*	*	*	*	*	*	*	*	*	*
AB571871	Thailand	Cattle	K	*	*	**-**	**-**	**-**	**-**	**-**	**-**	*	N	*	*	*	T	*	L	*	*	*	*	*
AB586125	Thailand	Water buffalo	*	*	*	**-**	**-**	**-**	**-**	**-**	**-**	*	*	*	*	*	*	*	*	F	*	*	*	*
AB617641	Syria	Cattle	*	*	*	**-**	**-**	**-**	**-**	**-**	**-**	*	*	*	E	*	*	*	*	*	*	*	*	*
KF192805	Egypt	Water buffalo	*	*	*	-	-	-	-	-	-	*	*	*	*	*	*	*	*	*	*	*	*	*
KF626638	South Africa	Cattle	*	A	*	**-**	**-**	**-**	**-**	**-**	**-**	*	*	V	*	*	*	D	*	*	S	*	*	*
KF626636	South Africa	Cattle	*	*	*	**-**	**-**	**-**	A	E	G	T	*	*	*	*	*	N	*	*	S	*	A	G
KF626634	South Africa	Cattle	*	*	*	**-**	**-**	**-**	A	E	G	*	*	*	*	*	*	N	*	*	S	*	A	G
KF626635	South Africa	Cattle	*	*	*	**-**	**-**	**-**	A	E	G	*	*	*	*	I	*	N	*	*	S	*	A	G
AB569301	Ghana	Cattle	*	*	*	A	E	G	A	E	G	*	*	*	*	I	*	N	*	*	S	*	A	G
AB569303	South Africa	Cattle	*	*	*	A	E	G	A	E	G	*	*	*	*	I	*	N	*	*	S	*	A	G
KF192806	Egypt	Cattle	*	*	R	A	E	G	A	E	G	*	*	*	*	I	*	N	*	*	S	*	A	G
KF192807	Egypt	Cattle	*	*	*	A	E	G	A	E	G	*	*	*	*	I	*	N	*	*	S	S	A	G
**KP347555**	Kenya	Cattle	*	*	*	A	E	G	A	E	G	*	*	*	*	I	*	N	*	*	S	*	A	G
**KP347556**	Kenya	Cattle	*	*	*	A	E	G	A	E	G	*	*	*	*	I	*	N	*	*	S	*	A	G
**KP347557**	Kenya	Cattle	*	*	*	A	E	G	A	E	G	*	*	*	*	I	*	N	*	*	S	*	A	G

Identical residues are indicated by stars (*). Numbers above the alignments represent the amino acid position taking *Babesia bovis* T2Bo (XM_001610418) as reference sequence. The sequences determined in this study are shown in bold-font

The identities among the *T. parva* p104 sequences ranged from 97.8 to 98.9 %. The Kenyan *T. parva* isolates detected were genetically different from *T. parva* isolates previously reported in Kenya. One of the *T. parva* isolates [KP347564] shared 99 % nucleotide identity with a previously published sequence from Kenya [AY034071]. However, the other two isolates [KP347565 and KP347566] shared 99 % nucleotide sequence identity with isolates from Zambia [AB739676 and AB739678], Zimbabwe [AY034070] and Kenya [AY034069]. Only one *T. parva* isolate [KP347565] was prevalent in Machakos farm while all the three isolates found in this study were detected in samples from Ngong farm. The identities among the four *T. orientalis* MPSP sequences of this study ranged from 86.5 to 99.5 %. The nucleotide sequences of three *T. orientalis* isolates [KP347560, KP347562 and KP347563] were conserved and shared 99 % sequence identity with *T. buffeli* [AB016278], a hemoparasite, previously isolated in Kenya. The other isolate [KP347561] shared 99 % sequence identity to the isolates from China [KJ020560 and AB571974], Thailand [AB562563] and Japan [AB218444].

### Phylogenetic analysis

**Fig. 1:**
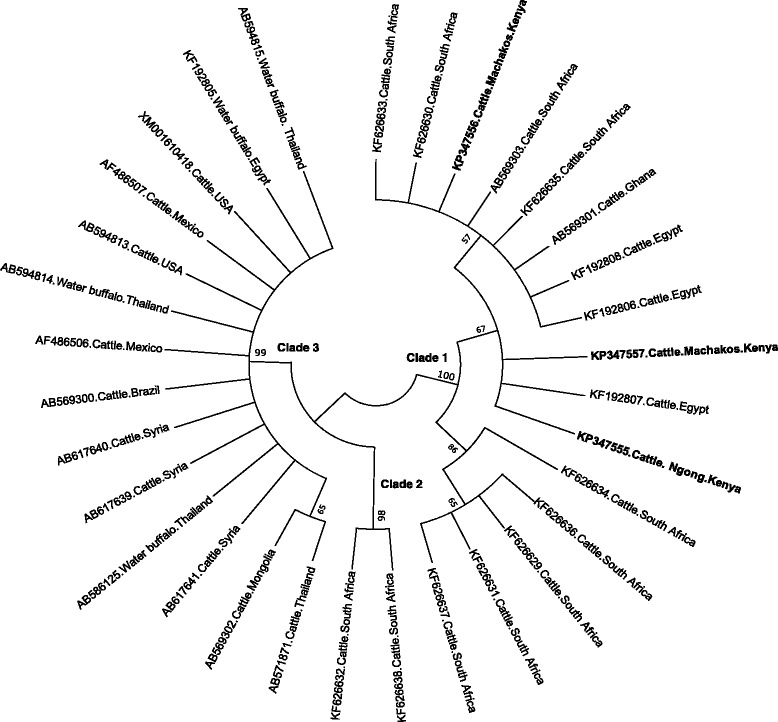
Unrooted phylogenetic tree of *Babesia bovis* SBP-4 gene. The tree was constructed with the maximum likelihood method using the Kimura 2 parameter model in the MEGA ver.6. The sequences determined in this study are shown in bold-font. Numbers on internodes indicate percentages of 1000 bootstrap replicates

**Fig. 2:**
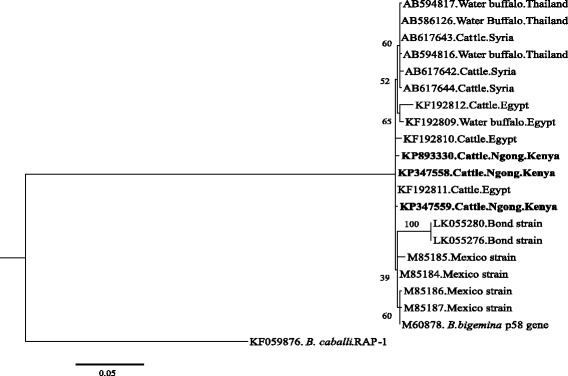
Phylogenetic analyses of *B. bigemina* RAP-1a gene sequences obtained from Kenyan cattle. *B. caballi* Rhoptry-associated protein-1(RAP1) gene was used as outgroup. The tree was constructed with the maximum likelihood method using the Kimura 2 parameter model in the MEGA ver.6. The sequences determined in this study are shown in bold-font. Numbers on the branches show percentages of 1000 bootstrap replications. The scale bar indicates estimated number of substitutions per site

**Fig. 3:**
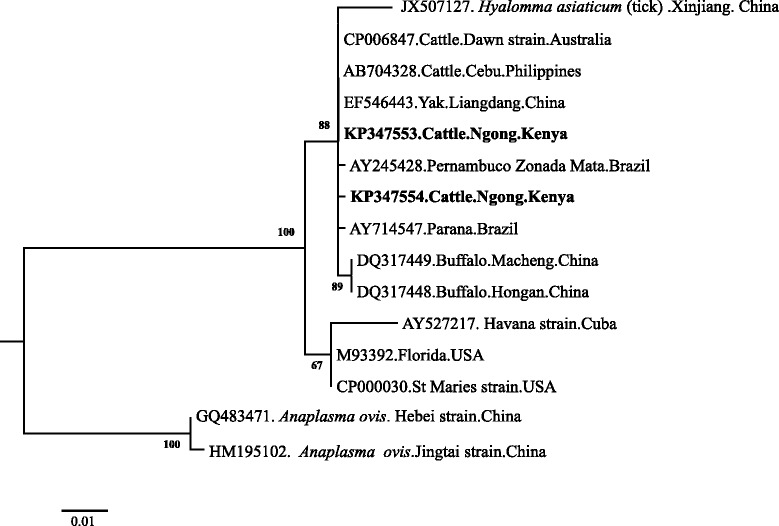
Phylogenetic analyses of *A. marginale* Msp 5gene sequences obtained from Kenyan cattle. *Anaplasma ovis* was used as outgroup. The tree was constructed with the maximum likelihood method using the Kimura 2 parameter model in the MEGA ver.6. The sequences determined in this study are shown in bold-font. Numbers on the branches show percentages of 1000 bootstrap replications. The scale bar indicates estimated number of substitutions per site

**Fig. 4:**
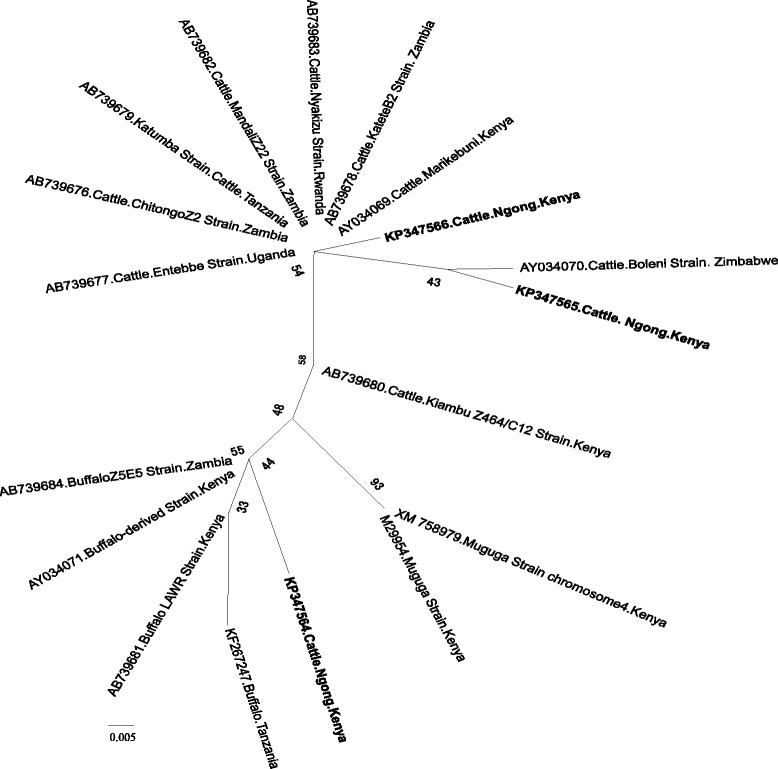
Unrooted phylogenetic tree showing relationships between *T.parva* isolates based on p104 gene. The tree was constructed with the maximum likelihood method using the John-Taylor Thornton with Gamma distribution (JTT + G) model in the MEGA ver.6. The sequences determined in this study are set in bold-font. Numbers on internodes indicate percentages of 1000 bootstrap replicates. The scale bar indicates estimated number of substitutions per site

**Fig. 5:**
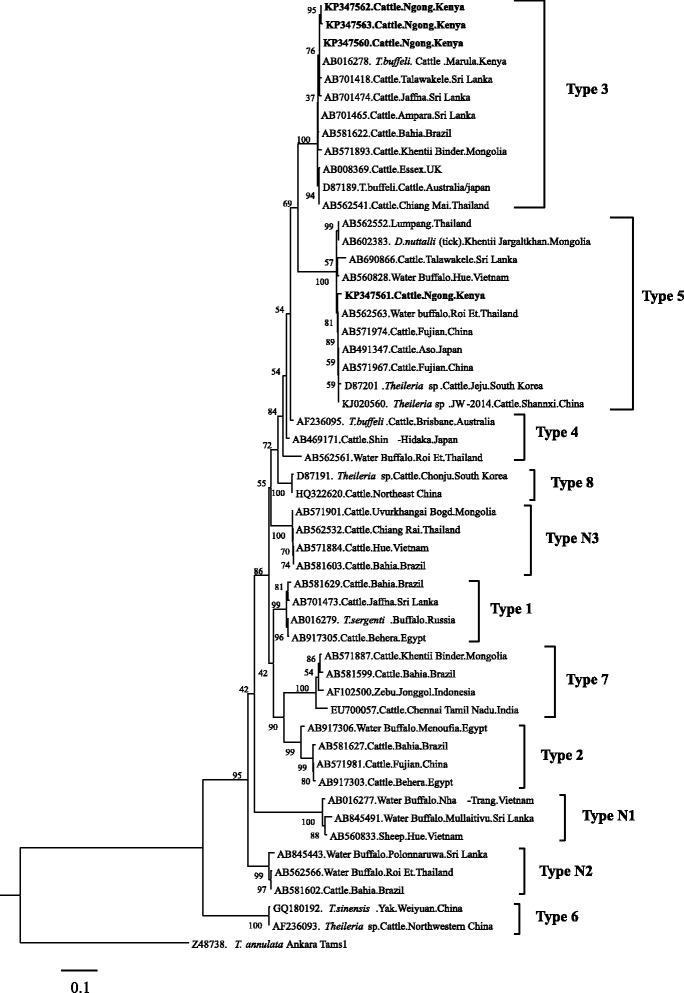
Phylogenetic analyses of *T. orientalis* MPSP gene sequences obtained from Kenyan cattle. The tree was constructed with the maximum likelihood method using the Tamura 3 parameter with Gamma distribution (T92 + G) model in the MEGA ver. 6. *T. annulata* (Ankara strain) merozoite surface antigen 1 gene (Tams1) was used as outgroup. The sequences determined in this study are shown in bold-font. Numbers on the branches show percentages of 1000 bootstrap replications. The scale bar indicates estimated number of substitutions per site

Phylogenetic analyses were done to determine whether the tick-borne pathogens are genetically diverse within different geographical regions of the world. Analysis based on SBP-4 gene grouped the Kenyan *B. bovis* isolates in the same clade (Clade 1) as Egyptian, Ghanaian and South African isolates (Fig. 1). The other *B. bovis* isolates from Thailand, Syria, Mexico, Brazil, Mongolia and United States of America (USA) were grouped in a separate clade. The Kenyan *B. bigemina* isolate belonged to the same clade as the isolates from Egypt, Thailand, Syria and Mexico (Fig. 2). A further phylogeny using the Msp5 gene grouped the Kenyan *A. marginale* isolates in the same clade as the isolates from China, Australia, Brazil and The Philippines (Fig. 3). However, isolates from USA and Cuba were grouped in a different clade. For *T. parva*, the [KP347565] and [KP347566] isolates were closely related to the cattle-derived genotypes while the other isolate [KP347564] was related to the buffalo-derived *T. parva* genotypes (Fig. 4). The polymorphism of *T. parva* p104 nucleotide sequences is shown in an additional file [see Additional file 3]. The phylogenetic analysis based on *MPSP* gene of *T. orientalis/sergenti/buffeli* grouped three of the isolates of this study [KP347560, KP347562 and KP347563] in the same clade and these isolates were classified as *MPSP* type 3. The divergent isolate [KP347561] belonged to a separate clade and was identified as *MPSP* type 5 (Fig. 5).

## Discussion

This study was done to determine the genetic diversities as well as molecular epidemiology of *B. bovis*, *B. bigemina, Theileria* spp. and *A. marginale* isolates of cattle raised in Machakos and Ngong districts of Kenya. Such information is critical for controlling and preventing infections caused by these pathogens, which leads to loss of livelihoods of many livestock owners [30]. The causative agents of babesiosis, theileriosis, and anaplasmosis were prevalent in the two farms surveyed. These findings are consistent with previous reports ranking tick-borne pathogens as important causes of diseases in Kenyan cattle [6–8, 31, 32]. Though *Theileria* spp. has always been reported to be the most prevalent hemoparasite in Kenya [8, 11, 12], this was not the case in Ngong farm as *Babesia* species were the most prevalent pathogens. However, *Theileria s*pecies were more prevalent in Machakos farm consistent with the previous studies in Kenyan cattle [8, 11, 12]. *A. marginale* was the least prevalent in both farms suggesting that it may not be one of the most prevalent tick-borne diseases in these regions.

The prevalence of *B. bovis* was higher than that of *B. bigemina* in Machakos and vice-versa in Ngong farm indicating that the epidemiology of babesiosis in the two farms may be different. The prevalence of *B. bigemina* reflects the distribution of its vectors, *Rhipicephalus evertsi* and *R. decoloratus* [1] which are both present in Machakos and Ngong districts [8, 33]. *B. bigemina* is the main cause of bovine babesiosis in Kenya [8, 34] and its predominance in Ngong farm is not surprising. The low prevalence of *B. bigemina* in Machakos farm could be explained by lower exposure to tick vectors. The detection of *B. bovis* was unexpected since none of its vectors namely, *R. microplus, R. geigyi* and *R. annulatus* [1] have been reported in the two districts. Our results, therefore indicate that *B. bovis* has been overlooked or is becoming endemic in Machakos and Ngong districts. The competition between *R. microplus* and *R. decoloratus* may explain why *B. bigemina* is more widespread than *B. bovis* in Africa [1]. Hence, the higher prevalence of *B. bovis* in Machakos farm suggests changes in tick distribution. Uncontrolled animal movement that is common in Kenya [34] or changes in ecological pattern may have contributed to the thriving of *B. bovis* tick-vectors.

This study revealed higher prevalences of *B. bovis* and *B. bigemina* than those previously observed in Western Kenya [16, 17]. This may not be surprising as the studies were done in different ecological areas. *B. bovis*, *B. bigemina* isolated in the two farms were genetically conserved and closely related to isolates from other African countries. *B. bovis* SBP-4 sequences from Kenya were consistent with the findings of [35] which indicated the existence of a “fingerprint” discriminating *B. bovis* isolates of African cattle from others. However, *B. bovis* SBP-4 phenogram showed the existence of an “intermediate” clade (Clade 2, Fig. 1) hosting sequences from South Africa which despite being isolated from cattle were not bearing the discriminatory “fingerprint”. Hence, further studies on *B. bovis* SBP-4 gene in other African countries are needed for clarification of the scope, the origin and impact on the parasite of this “fingerprint”.

The detection of *A. marginale* DNA in both farms is consistent with previous studies which reported antibodies to *A. marginale* in Kenyan cattle [8, 12–14]. The sequence identity of the two *A. marginale* genotypes detected in this study suggests that the parasite isolates circulating in Kenya may be genetically conserved. However, further studies are required to unravel the genetic diversity of the parasite isolates.

Although this study detected *Theileria* species in both farms, the samples analyzed here were not large enough and elaborate studies will be needed to determine the full extent of the infections in Kenya. Many *Theileria* species including *T. parva*, *T. taurotragi, T. mutans* and *T. velifera* were detected in both farms. However, *T.orientalis/sergenti/buffeli*, *T. ovis* and *Theileria* sp*.* (buffalo) were detected only in Ngong farm. Cattle are the natural host of *T. parva*, *T. taurotragi*, *T. mutans* and *T. velifera. R.appendiculatus,* which transmits *T. parva* and *T. taurotragi* as well as *Amblyomma variegatum,* the vector for *T. mutans* and *T. velifera* are known to exist in the districts surveyed [8, 33]. Therefore, the presence of these tick vectors may explain the occurrences of the hemoparasites in the farms. *T. ovis* is known to infect small ruminants [36] while *T. buffeli* and *Theileria* sp*.* (buffalo) infect African Cape buffalo (*Syncerus caffer)* [37]. The detection of buffalo-*Theileria* isolates in Ngong farm-cattle may be attributed to their transmission by ticks and interaction of these cattle with nomadic Maasai cattle. These Maasai cattle usually graze together with wildlife including buffaloes. Likewise, Maasai cattle are generally kept with flocks of small ruminants and therefore can be a source of *T. ovis*–infected ticks for Ngong farm-cattle. Such accidental *Theileria* infections in cattle have previously been reported in wildlife–domestic animals interface and in areas where there is high animal movement in Kenya [15–17]. Before this study, only one *MPSP* allele of *T. sergenti/buffeli/orientalis* had been isolated in Kenya [38, 39]. The polymorphism of the *T. orientalis MPSP* type 3 isolates identified and the description for the first time of *T. orientalis MPSP* type 5 indicate that at least two strains of this benign *Theileria* parasite are present in Kenya. None of the *T. orientalis* MPSP alleles identified in Kenya have ever been associated to disease outbreaks. However, in Ethiopia [40], Burundi [41], India [42], Australia [43–45] and New Zealand [46], some *T. orientalis* strains have caused disease outbreaks. Hence, *T. orientalis* complex should not be ruled out as a probable cause of disease, particularly for crossbred and exotic breeds dairy cattle reared in Kenya.

*T. parva* was the most frequent *Theileria* in the two farms. Although they were positive for both *Theileria* 18S rRNA and *T. parva* p104 nPCRs, sequencing of the *Theileria* 18S rRNA amplicons of 35 samples from Ngong farm and 11 samples from Machakos farm, did not show a sequence specific to *T. parva*. This is probably due to low levels of *T. parva*-parasitemia and to several *Theileria* species co-occurring in the same animal. In the current study, genus specific primers were used to amplify *Theileria* 18S rRNA and obtained products therefore contained amplicons from several species. Only amplicons derived from species with abundant DNA were reflected in the sequencing chromatogram. Hence, *T. parva* 18S rRNA was not identified in some samples because it was outnumbered by the 18S rRNA gene of other *Theilera* species. East Coast fever (ECF) caused by *T. parva* is the most important tick-borne disease in Kenya, and “immunization” against it is common [6, 47, 48]. The carrier state is particularly important for this parasite as it contributes to and may be necessary for maintenance of immunity against overt disease [18, 36]. Most of *T. parva* positive animals in this study seemed to be carriers and previous studies [49–51] suggested that such status could have been induced by previous “immunization” as well as natural infection. *T. parva* prevalences obtained were lower than the 67 % observed in Marula, Rift Valley [15], similar to the values recorded by [18] but higher than the RLB data from Western Kenya [16, 17]. The detection of *T. parva* isolate in Machakos farm and not the buffalo-derived genotypes suggested that these cattle are only exposed to the cattle-derived *T. parva.* The identification of buffalo-derived genotypes in Ngong farm corroborates previous reports of the occurrence of buffalo-derived *T. parva* in cattle in Kenya [33, 34].

All the cattle in this study appeared healthy, although pathogenic hemoparasites (*B. bovis*, *B. bigemina*, *A. marginale* and *T. parva*) were detected in their blood. This absence of clinical disease in infected cattle may be attributed to a state of enzootic stability as described in previous reports [1, 3, 4]. The high rate of multiple infections in both farms, sometimes involving hemoparasites belonging to different genus may be explained by the presence of a range of tick-vectors that exist in the same ecosystem. Benign *T. taurotragi*, and *T. mutans* detected in this study have been previously associated with morbidity and mortality in calves in Kenya [52, 53]. Perhaps further studies should explore the importance of these hemoparasites in Kenya with regard to *Theileria* infections in calves. SBP-4, RAP-1a, Msp5 and MPSP genes, and the corresponding PCR assays were exploited for the first time in Kenya. Our findings confirm the value of these assays [21, 22, 24, 35, 45] and suggest that they can be used to improve hemoparasites detection in Kenya.

## Conclusions

This study has confirmed the occurrences of a range of genetically diverse tick-borne hemoparasites in farms located in two districts of Kenya. The detection, prevention and control of these hemoparasites in cattle should consider their genotypes, the co-infective nature of these pathogens and the role of wildlife in the transmissions of the tick-borne parasites. Therefore, this study will provide a basis for further research on tick-borne hemoparasitic diseases and their molecular epidemiology in Kenya and other regions of the world.

## Additional files

Additional file 1: Table S1. Results of hemoparasites DNAs detection by nPCR. This table presents individual results for each parasite per study farm. (DOCX 21 kb) Additional file 2: Table S2. Theileria species detected in cattle from Ngong-farm and Machakos-farm in Kenya. This table summarizes the results of the identification of *Theileria* infections based *Theileria* spp. 18S RNA amplicons, *T. parva* p104 nPCR and *T. orientalis* MPSP PCR. (DOCX 25 kb) Additional file 3: Table S3. Nucleotide alignment of partial sequences of *T. parva* p104 gene. This table presents a multi-sequence alignment of *T. parva* p104 sequences which compares the sequences obtained in this study to those previously published. (XLSX 28 kb)

## Abbreviations

BLAST: Basic local alignment tool
DNA: Deoxyribonucleic acid
EDTA: Ethylenediaminetetraacetic acid
ECF: East coast fever
Msp5: Major surface protein 5
MPSP: Major piroplasm surface protein
NCBI: National center for biotechnology information
nPCR: Nested PCR
PCR: Polymerase chain reaction
RAP-1a: Rhoptry-associated protein 1a
SBP-4: Spherical body protein 4
18S rRNA: component of the small subunit of the ribosomal ribonucleic acid
Tams1: *T. annulata* merozoite surface antigen 1
