# Supplementary material for: Molecular detection and characterization of Babesia bovis, Babesia bigemina, Theileria species and Anaplasma marginale isolated from cattle in Kenya
Source: Parasit Vectors. 2015 Sep 30;8:496. doi: 10.1186/s13071-015-1106-9 (PMC4589125; doi:10.1186/s13071-015-1106-9)
Supplement: Additional file 1: Table S1. — Results of hemoparasites DNAs detection by nPCR. This table presents individual results for each parasite per study farm. (DOCX 21 kb) [file 13071_2015_1106_MOESM1_ESM.docx]

Table S1. **Results of hemoparasites DNAs detection by nPCR**

| Farm | No. of samples | Positive samples (%) | | | |
| --- | --- | --- | --- | --- | --- |
|  |  | *B. bovis* | *B. bigemina* | *Theileria* spp. | *A. marginale* |
| Ngong | 154 | 19 (12.3) | 65 (42.2**^a^**) | 52 (33.8) | 50 (32.5**^a^**) |
| Machakos | 38 | 9 (23.7) | 5 (13.2**^b^**) | 15 (39.5) | 6 (15.8**^b^**) |
| Total | 192 | 28 (14.6) | 70 (36.5) | 67 (34.9) | 56 (29.2) |

Unlike superscript letters (**a,b**) in the same column, denotes significant difference between samples (*P*< 0.05 ).
