# Supplementary material for: Molecular detection and characterization of Babesia bovis, Babesia bigemina, Theileria species and Anaplasma marginale isolated from cattle in Kenya
Source: Parasit Vectors. 2015 Sep 30;8:496. doi: 10.1186/s13071-015-1106-9 (PMC4589125; doi:10.1186/s13071-015-1106-9)
Supplement: Additional file 2: Table S2. — Theileria species detected in cattle from Ngong-farm and Machakos-farm in Kenya. This table summarizes the results of the identification of Theileria infections based Theileria spp. 18S RNA amplicons, T. parva p104 nPCR and T. orientalis MPSP PCR. (DOCX 25 kb) [file 13071_2015_1106_MOESM2_ESM.docx]

Table S2. ***Theileria* species detected in cattle from Ngong-farm and Machakos-farm in Kenya**

| *Theileria* species detected | Number of positive samples | |
| --- | --- | --- |
|  | Ngong-farm | Machakos-farm |
| *T. parva* | 11**^a^** + 35**^b^** | 1**^a^** + 11**^b^** |
| *T. velifera* | 16 | 9 |
| *T. taurotragi* | 11 | 4 |
| *T. mutans* | 11 | 3 |
| *T. orientalis/buffeli/sergenti* | 3**^c^** | 0 |
| *T. ovis* | 3 | 0 |
| *Theileria sp. ex Syncerus caffer* | 1 | 0 |

**^a^**: Number of samples in which *T. parva* 18S ribosomal RNA and *T. parva* p104 genes were detected.

**^b^**: Number of samples that were negative for *T. parva* 18S rRNA but found to be positive for *T. parva* p104 DNA.

**^c^**: Number of samples in which *T. orientalis* 18S ribosomal RNA and *T. orientalis* MPSP genes were detected.

^No letter^ : Number of samples in which the 18S ribosomal RNA gene of the corresponding *Theileria* species ( species on the same line of the table) were detected.
